# Digital monitoring of disease activity in relapsing–remitting multiple sclerosis

**DOI:** 10.1007/s00415-026-13685-5

**Published:** 2026-03-06

**Authors:** P. C. G. Molenaar, D. J. de Jong, S. N. Hof, P. van Oirschot, I. G. Bucur, K. H. Lam, B. Moraal, T. M. Heskes, V. de Groot, B. M. J. Uitdehaag, B. A. de Jong, J. W. R. Twisk, E. M. M. Strijbis, J. Killestein

**Affiliations:** 1 MS Center Amsterdam, Neurology, Amsterdam Neuroscience, Vrije Universiteit Amsterdam, Amsterdam UMC Location VUmc, Amsterdam, The Netherlands; 2 MS Center Amsterdam, Anatomy and Neurosciences, Amsterdam Neuroscience, Vrije Universiteit Amsterdam, Amsterdam UMC Location VUmc, Amsterdam, The Netherlands; 3 Sherpa B.V., Nijmegen, The Netherlands; 4 Institute for Computing and Information Sciences, Radboud University, Nijmegen, The Netherlands; 5 MS Center Amsterdam, Radiology and Nuclear Medicine, Amsterdam Neuroscience, Vrije Universiteit Amsterdam, Amsterdam UMC Location VUmc, Amsterdam, The Netherlands; 6 Department of Rehabilitation Medicine, Amsterdam UMC, Amsterdam, The Netherlands; 7 Department of Epidemiology and Data Science, Amsterdam UMC, Amsterdam, The Netherlands; 8 Amsterdam UMC, Location VUmc Polikliniek Neurologie, Attn. MS Center Amsterdam, PO Box 7057, 1007 MB Amsterdam, The Netherlands

**Keywords:** Relapsing–remitting multiple sclerosis, Disease activity, Digital biomarkers, MRI, Gadolinium-enhancing lesions

## Abstract

**Background:**

Digital monitoring shows promise for detecting disease activity in people with relapsing–remitting multiple sclerosis (PwRRMS). Here, we study associations between digital biomarkers for cognition and walking ability and radiological disease activity.

**Methods:**

In a prospective, 1-year cohort study, PwRRMS performed the smartphone-based symbol digit modalities test (sSDMT) and 2-min walk test (s2MWT) on weekly basis. MRIs and the clinical SDMT (cSDMT), expanded disability status scale (EDSS), timed 25-foot walk (T25FW) and nine-hole peg test (NHPT) were collected at baseline and every 3 months. Associations were determined using logistic generalized estimating equations. For the digital measures, associations were also analyzed using a hybrid model and were repeated with values from 6 weeks before and after MRI.

**Results:**

We included 57 PwRRMS. The sSDMT was negatively associated with contrast-enhancing lesions (CELs) (OR_overall_ 1.80, 95% CI 1.12–2.91), predominantly caused by variation within individuals (OR_within-subjects_ 4.37, 2.05–9.33), with a similar relation using sSDMT values 6 weeks prior to MRI (OR_overall_: 1.92, 0.947–3.90, OR_within-subjects_: 13.7, 1.74–107). The negative association between s2MWT and CELs (OR_overall_ 1.20, 1.04–1.38) was caused equally by variation within and between individuals. All clinical measures were negatively associated with CELs: T25FW (OR_overall_ 2.23, 1.50–3.32), EDSS (OR_overall_ 1.49, 0.932–2.39), cSDMT (OR_overall_ 1.20, 1.02–1.42) and NHPT (OR_overall_ 1.15, 1.04–1.27).

**Discussion:**

Digital biomarkers show to be capable of measuring changes in individuals when inflammation is detectable on MRI, with the sSDMT additionally capturing changes 6 weeks prior to the MRI, suggesting that early identification of inflammation using these biomarkers may be possible.

**Supplementary Information:**

The online version contains supplementary material available at 10.1007/s00415-026-13685-5.

## Introduction

In multiple sclerosis (MS) care, disease activity is monitored through regular MRI scans and through clinical assessments in case of new or acutely worsening symptoms [1, 2]. As disease activity frequently occurs in the absence of a noticeable worsening of clinical symptoms, with clinical visits and MRI scans being planned according to protocolized time intervals, the onset of inflammatory activity is commonly missed and may remain undetected for months to years [2, 3]. Consequently, adjustments in disease-modifying therapies (DMTs) to prevent future inflammation and progression are delayed [1]. Digital biomarkers might be a useful addition to clinical care and research to detect disease activity in between scans.

Digital monitoring enables frequent, objective, and real-world data collection on the functioning of people with relapsing–remitting MS (PwRRMS) in their own environment. As digital biomarkers aim to capture both short-term changes and functioning over more extensive periods of time, they might be able to capture subtle changes in clinical functioning due to MS-related inflammation and progression which are missed by less frequently administered conventional clinical measures. Moreover, digital biomarkers are easy to capture without the necessity to visit an outpatient clinic, which lowers the burden for PwRRMS and the healthcare system [4–7]. Examples of such tools include the MS sherpa® smartphone-based symbol digit modalities test (sSDMT) for monitoring cognitive functioning [5, 7] and the smartphone-based 2-min walk test (s2MWT) for monitoring walking ability [4, 6]. The reliability, construct validity, and reproducibility parameters of these digital biomarkers have been established in previous research [4–7]. Concurrent validity and responsiveness have been demonstrated using conventional clinical measures for monitoring progression in MS, consisting of the clinical symbol digit modalities test (cSDMT) for the sSDMT [7, 8] and the expanded disability status scale (EDSS) and timed 25-foot walk (T25FW) for the s2MWT [6, 8]. In addition, the sSDMT has shown cross-sectional correlations with thalamic volume and lesion volume [9]. The nine-hole peg test (NHPT) has not been previously used to assess the validity of these digital biomarkers. Although digital biomarkers hold promise to monitor clinical worsening due to inflammatory disease activity [10], they have not been validated for this purpose yet. If digital biomarkers are able to capture subtle clinical worsening in the presence of radiological disease activity, they might be used as a marker to detect disease activity. Theoretically, they could then help caregivers decide to advance or postpone MRI scans outside of the standard protocol.

Here, we study associations between digital biomarkers for cognition and walking ability (sSDMT and s2MWT) and radiological disease activity (presence of contrast-enhancing lesions) in PwRRMS over time. Moreover, we determine if conventional measures (EDSS, T25FW, cSDMT, NHPT) for monitoring disability in MS are also associated with radiological disease activity.

## Methods

### Population and data collection

PwRRMS were included from the ‘Assessing fatigue, disease activity and progression through smartphone surveillance in multiple sclerosis’ (APPS-MS) study, a 1-year prospective cohort study at the MS Center Amsterdam, Amsterdam University Medical Center from August 2018 to January 2021. The study received ethical approval (METc VUmc 2017.576). Written informed consent was collected prior to inclusion.

The study design and prior analyses regarding clinimetric properties, as well as associations with radiological and clinical measures in this cohort, were reported previously [4–7, 9]. In brief, included participants had a definite diagnosis of RRMS according to the McDonald 2017 criteria [11], were aged between 18 and 65 years and reported regular use of a smartphone. PwRRMS with an EDSS ≥ 7.5, changes in MS medication 2 months prior to screening, clinically relevant visual disturbances and a confirmed diagnosis of current and relevant mood, behavioral or sleeping disorders were excluded from this study at the discretion of the investigator.

**Fig. 1:**
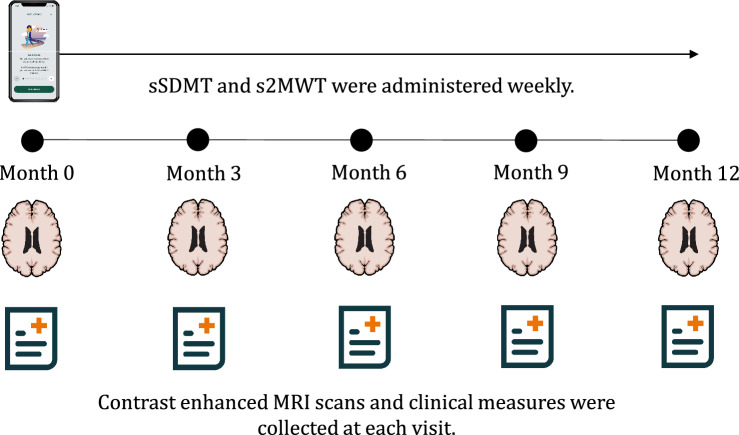
Study schedule. Clinical monitoring consisted of the expanded disability status scale, timed 25-foot walk, symbol digit modalities test, and nine-hole peg test. *MRI* magnetic resonance imaging, *sSDMT* smartphone-based symbol digit modalities test; *s2MWT* smartphone-based 2-min walk test. Images from pixabay.com

During 1-year follow-up, participants performed the sSDMT and s2MWT tests weekly using the MS sherpa^®^ application. In the first 4 weeks of the study, participants performed both tests every 3 days in the morning and evening (5). EDSS, T25FW (mean of two trials), cSDMT and NHPT (mean of two trials for each hand), as well as MRI scans including post-contrast T1 sequences, were collected during clinical visits at baseline and subsequently every 3 months. Radiological disease activity was defined as the presence of at least one contrast-enhancing lesion on MRI (yes/no), evaluated by an experienced neuroradiologist (BM). Figure 1 shows the study schedule.

### Digital biomarkers and modeling

**Fig. 2:**
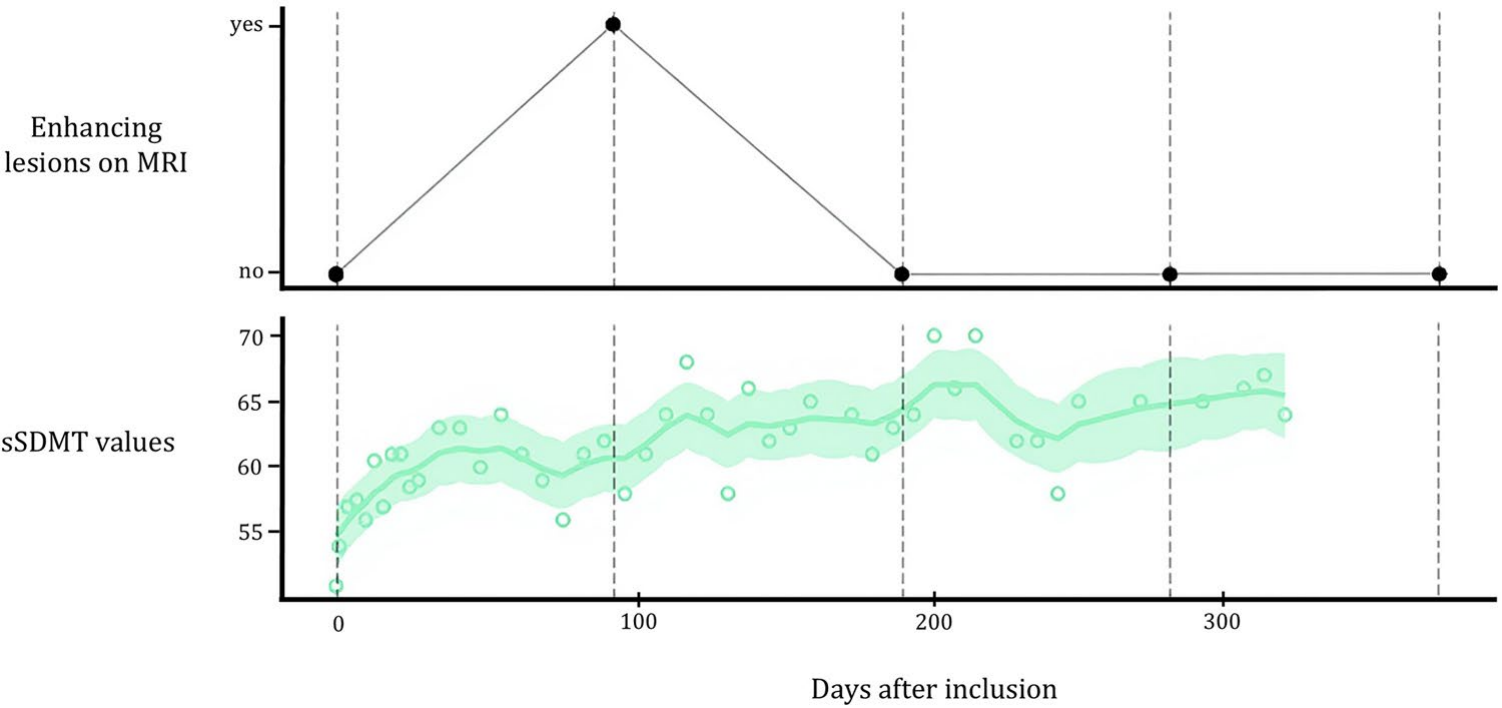
sSDMT curve fitting for an individual with radiological disease activity. Upper panel shows the presence of gadolinium-enhancing lesions per visit. Lower panel shows the raw measurements (dots), the local linear trend model fit (smooth curve) and 95% confidence intervals (band around curve), with the sSDMT score in points on the y-axis. *sSDMT* smartphone-based symbol digit modalities Test, *MRI* magnetic resonance imaging

The MS sherpa^®^ (Sherpa, Nijmegen, The Netherlands) app enables PwRRMS to self-administer the s2MWT and sSDMT, using their own smartphone device. The sSDMT measures cognition through a 90-s trial in which digits are matched to symbols (score is number of correct digit-symbol combinations) [5, 7], whereas the s2MWT measures walking ability by recording the distance covered during a 2-min trial (score is in meters) [4, 6]. For both the sSDMT and the s2MWT, lower scores correspond to higher rates of disability [5, 6]. Given the high frequency in which the sSDMT and s2MWT are administered, they are likely susceptible to measurement error due to physiological variation over short periods of time. Therefore, in line with prior research, underlying ‘true’ scores (i.e., level values) based on the sSDMT and s2MWT ‘raw’ measurements (i.e., test scores collected from the persons smartphone device) were estimated using a local linear trend model (LLTM) [6, 7]. This state space model enables the estimation of the underlying level values via weighted aggregation of the raw measured scores, taking modeling assumptions on the measurement error of individual measurements into account, resulting in more stable results [12]. Ultimately, this enables the estimation of level values for days on which no tests were actually performed. Moreover, a smoothed curve and a 95% confidence interval band around this curve are modeled to reflect the uncertainty in the model estimates [6, 7]. For the analyses in this study, only level values with a raw measurement within 14 days from the investigated timepoint were used. An example of the LLTM curve fitting of the sSDMT of one individual is shown in Fig. 2.

### MRI acquisitions and analyses

MRI acquisition was performed on a 3 T GE Discovery MR750 (GE Healthcare, Milwaukee, USA) with an 8-channel head coil. All scans in this study were acquired using the same scanner. The scan protocol included a pre- and post-gadolinium contrast-enhanced axial 2D-T1-weighted image (repetition time 600 ms, echo time 8 ms, flip angle 125°, field of view 250 mm, matrix 512 × 512 mm^2^, slice thickness 5.0 mm) on all visits.

### Statistical analyses

Descriptive analyses were conducted to characterize the baseline demographics and the digital and clinical scores for each study visit. For continuous variables, means with standard deviations (SD) were reported, in case of a skewed distribution, and for ordinal variables, medians with interquartile ranges (IQR) were reported. Overall associations between radiological disease activity (dependent variable) and digital biomarkers (independent variable) over time were determined through logistic generalized estimating equation (GEE) analyses with an exchangeable correlation structure, using data from the five study visits. To disentangle whether the association between radiological disease activity and digital biomarkers is due to variation within or between individuals, additional analyses using a logistic hybrid model were conducted. Within this model, the between-subject association is determined using the individual mean value over time as the independent variable, whereas the within-subject association is determined using the deviation score as the independent variable (i.e., the difference between observations and the individual mean value) [13]. We repeated these analyses with digital biomarker level values from 6 weeks prior and 6 weeks after the MRI as independent variable to explore their relation with radiological disease activity over a broader time interval. To further explore the variation within individuals across different timepoints, the within-subject association of these digital biomarkers with radiological disease activity was assessed using digital biomarker level values from 10 weeks prior to 10 weeks after the MRI scan, with 2-week lags. The odds ratios as function of the time lags were visualized with a forest plot [14]. To determine the association between conventional measures for monitoring disability in MS, GEE analyses were performed with the EDSS, T25FW, cSDMT, and NHPT scores on all five study visits as independent variables. These results, along with the results from the GEE analyses with the digital biomarker level values at the day of the MRI on all five study visits, were visualized using a forest plot.

All associations are described as odds ratios (ORs) with 95% confidence intervals and are corrected for sex and disease duration from diagnosis. ORs were calculated for 5, 10, and 5 units of change on the sSDMT, s2MWT, and cSDMT, respectively, to reflect ORs for a more substantial amount of change [15]. As the direction of scores that correspond with higher levels of disability is not the same for all included measures, we calculated inverse ORs of the sSDMT, s2MWT, and cSDMT. Subsequently, ORs exceeding 1 indicate that higher levels of disability are associated with the presence of CELs [16]. Prior to our analyses, we performed a missing data analysis, which is described in the supplementary materials. All analyses were performed in RStudio version 4.2.1 and IBM SPSS Statistics 28.

## Results

We included 57/61 (93%) participants from the APPS-MS study of which both MRI data and app data on at least one of the digital biomarkers on at least one of the five visits were available. For included individuals, sSDMT level values were available on 195/285 (68%) visits, while s2MWT data were available on 159/285 (56%) visits. The baseline demographical descriptives, as well as the descriptives of the digital and clinical measures for each clinical visit, are presented in Table 1. A total of 18 individual participants had at least one contrast-enhancing lesion during at least one of the study visits, of which none had a relapse or were treated with methylprednisolone in the 3 months prior to and during the study. The missing data analysis did not show relevant differences between participants with complete data and participants with missing data, as shown in the supplementary materials.

**Table 1:** Baseline demographical descriptives, as well as digital and clinical scores for each clinical visit

	Baseline	Month 3	Month 6	Month 9	Month 12
Participants (n)	57	–	–	–	–
Age (years)		–	–	–	–
Mean (SD)	42 (9.5)				
Sex		–	–	–	–
Male	10 (18%)				
Female	47 (82%)				
Disease duration from diagnosis (years)		–	–	–	–
Median [Q1-Q3]	5.1 [2.8–11]				
Participants with enhancing lesions					
Yes	13	5	4	5	3
No	41	46	35	35	32
s2MWT (meters)					
Mean (SD)	125 (38.9)	140 (32.9)	140 (36.5)	145 (41.2)	148 (42.9)
sSDMT (points)					
Mean (SD)	49.5 (6.38)	52.6 (7.34)	53.8 (7.29)	54.0 (7.38)	53.4 (7.64)
EDSS (points)					
Median [Q1-Q3]	3.0 [2.5–3.5]	3.0 [2.5–4.0]	2.5 [2.0–4.0]	3.0 [2.5–4.0]	3.0 [2.0–4.0]
T25FW (s)					
Mean (SD)	4.73 (1.46)	4.54 (1.02)	5.09 (2.24)	4.81 (1.10)	4.89 (1.33)
cSDMT (points)					
Mean (SD)	56.0 (9.23)	59.8 (9,89)	60.1 (11.3)	63.6 (12.7)	64.0 (12.0)
NHPT (s)					
Median [Q1-Q3]	20.3 [18.1–21.9]	19.8 [18.4–22.5]	19.5 [18.6–21.3]	19.8 [18.4–10.8]	19.6 [18.3–21.6]

For non-normally distributed, continuous variables, median values are shown

*Enhancing lesions* presence of at least one contrast-enhancing lesion on MRI scan, *sSDMT* smartphone-based symbol digit modalities test, *s2MWT* smartphone-based 2-min walk test, *EDSS* expanded disability status scale, *T25FW* timed 25-foot walk, *cSDMT* symbol digit modalities test, *NHPT* nine-hole peg test

**Fig. 3:**
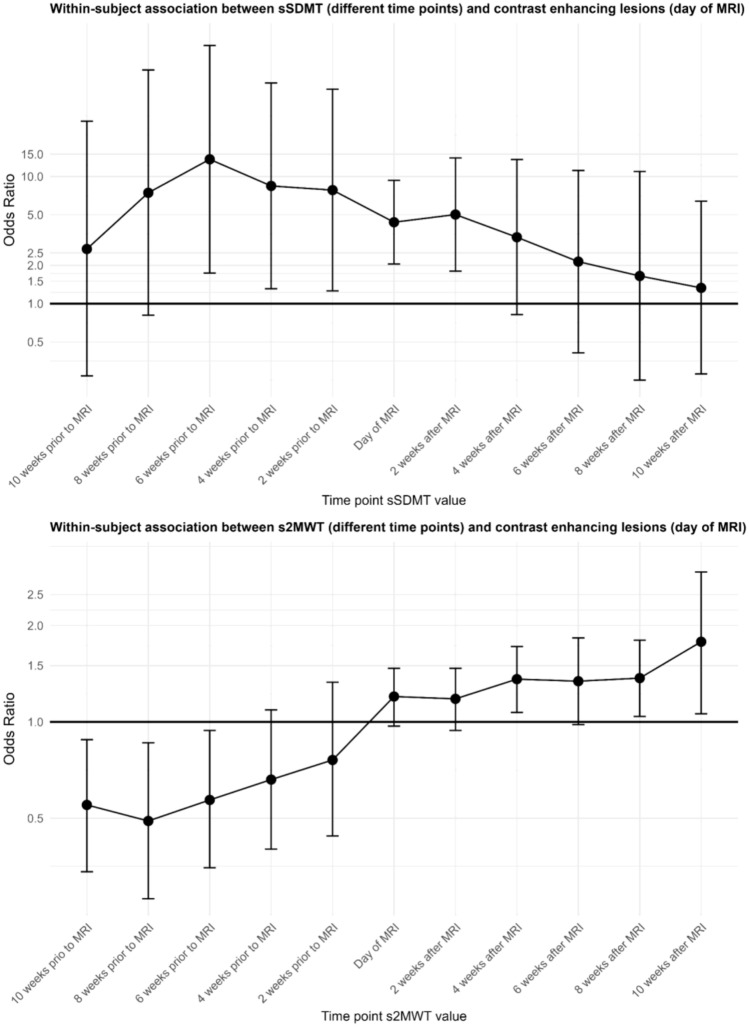
Within-subject associations of both digital biomarkers with contrast-enhancing lesions over time, using digital biomarker level values from 10 weeks prior to 10 weeks after the MRI scan. All associations are expressed in odds ratios with 95% confidence interval bands and are corrected for sex and disease duration from diagnosis. Odds ratios > 1 indicate that higher levels of disability are associated with the presence of contrast-enhancing lesions. We report odds ratios per 5-point decrease in sSDMT and per 10-m decrease in s2MWT. *CI* confidence interval; *MRI* magnetic resonance imaging; *sSDMT* smartphone-based symbol digit modalities test; s2MWT smartphone-based 2-min walk test

**Fig. 4:**
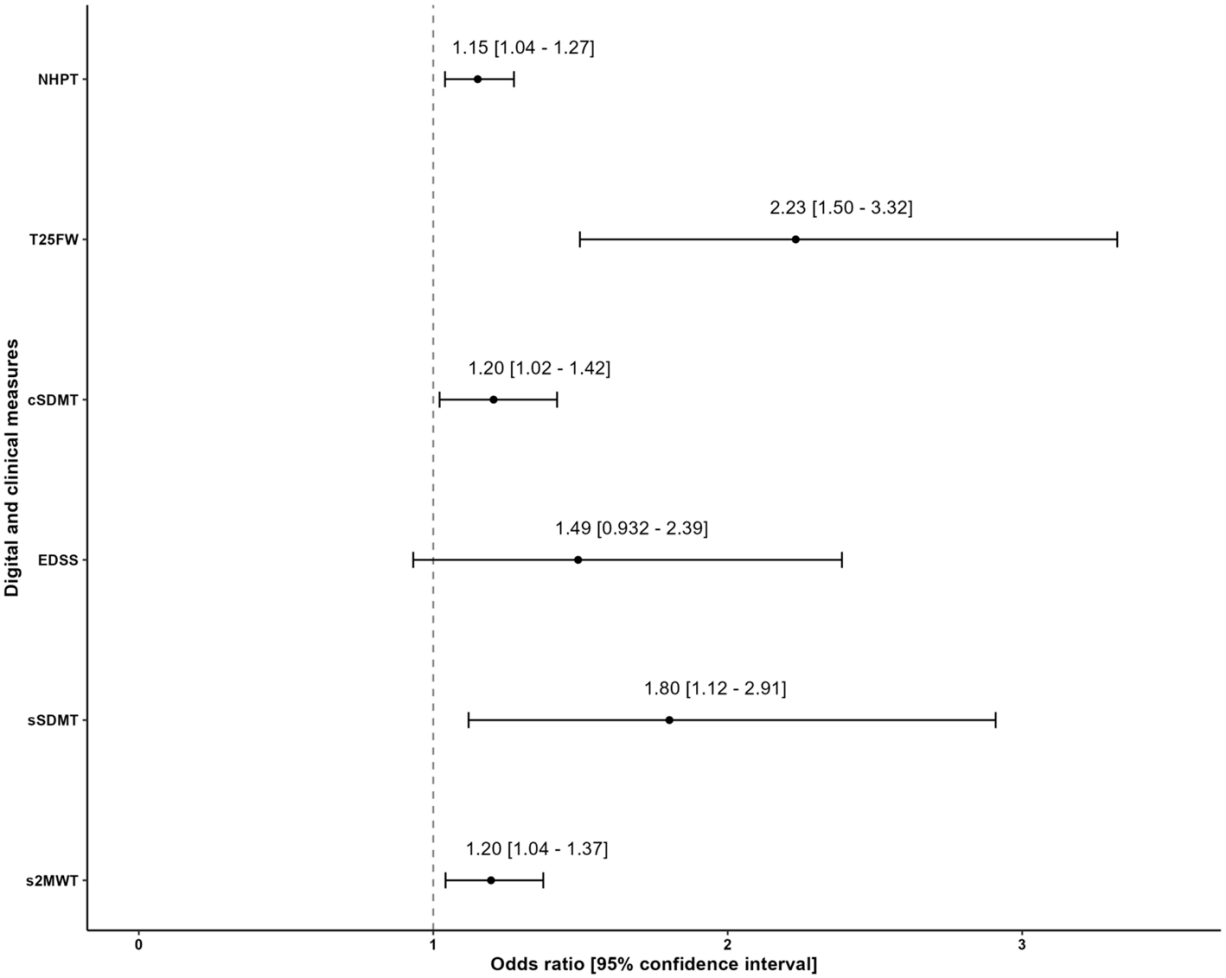
Overall associations between contrast-enhancing lesions and both digital and clinical measures on the day of the MRI, expressed in odds ratios with 95% confidence intervals. Results are corrected for sex and disease duration from diagnosis. Odds ratios > 1 indicate that higher levels of disability are associated with the presence of contrast-enhancing lesions. We report odds ratios per 5-point decrease in sSDMT and cSDMT, and per 10-m decrease in s2MWT. We report odds ratios per 1-s increase in NHPT and T25FW, and 1-point increase in EDSS. *NHPT* nine-hole peg test; *T25FW* timed 25-foot walk; *cSDMT* symbol digit modalities test, *EDSS* expanded disability status scale; *sSDMT* smartphone-based symbol digit modalities test; *s2MWT* smartphone-based 2-min walk test

Table 2 shows that contrast-enhancing lesions (CELs) were negatively associated with both the sSDMT (OR_overall_ = 1.80, 95% CI 1.12–2.91) and the s2MWT (OR _overall_ = 1.20, 95% CI 1.04–1.38) over time. The results from our hybrid model show that the association between CELs and the sSDMT was primarily caused by variation within individuals (OR_within-subjects_ = 4.37, 95% CI 2.05–9.33). This predominant within-subject association was not found between CELs and the s2MWT, as this relation shows to be caused by both variation within individuals (OR_within-subjects_ = 1.20, 95% CI 0.973–1.47) and variation between individuals (OR_between-subjects_ = 1.20, 95% CI 0.986–1.45). Moreover, our results show that the sSDMT is already negatively associated with CELs when using level values from 6 weeks prior to the MRI scan (OR_overall_ = 1.92, 95% CI 0.947–3.90), which was also caused by variation within individuals (OR_within-subjects_ = 13.7, 95% CI 1.74–107). Both this overall and within-subject association diminished 6 weeks after the MRI scan (OR_overall_ = 1.35, 95% CI 0.819–2.24, OR_within-subjects_ = 2.14, 95% CI 0.409–11.2). Figure 2 shows LLTM curve fitting of the sSDMT for an individual with radiological disease activity during the study period. The s2MWT does not show an overall association with CELs using level values from 6 weeks prior to the MRI scan (OR_overall_ = 1.05, 95% CI 0.820–1.34), but does show an extended association with CELs when using level values from 6 weeks after the MRI scan (OR_overall_ = 1.23, 95% CI 1.04–1.46). Figure 3 shows the within-subject association of both digital biomarkers with CELs on different timepoints, using digital biomarker level values from 10 weeks prior to 10 weeks after the MRI scan. Figure 4 visualizes the negative associations between CELs and clinical measures and digital biomarkers at the day of the MRI.

**Table 2:** Associations between radiological disease activity and digital biomarkers

	sSDMT^a^ Odds ratios (95% CI)	s2MWT^a^ Odds ratios (95% CI)
**Day of MRI**^**a**^	1.80 (1.12–2.91)	1.20 (1.04–1.38)
*Within subjects*^*b*^	4.37 (2.05–9.33)	1.20 (0.973–1.47)
*Between subjects*^*b*^	1.33 (0.737–2.39)	1.20 (0.986–1.45)
**6 weeks prior to MR**I^**c**^	1.92 (0.947–3.90)	1.05 (0.820–1.34)
*Within subjects*^*d*^	13.7 (1.74–107)	0.573 (0.350–0.937)
*Between subjects*^*d*^	1.33 (0.538–3.34)	1.18 (0.89–1.55)
**6 weeks after MRI**^**c**^	1.35 (0.819–2.24)	1.23 (1.04–1.46)
*Within subjects*^*d*^	2.14 (0.409–11.2)	1.34 (0.982–1.83)
*Between subjects*^*d*^	1.32 (0.797–2.18)	1.21 (0.982–1.49)

Associations between contrast-enhancing lesions and both digital biomarkers, expressed in odds ratios with 95% confidence intervals. Results are corrected for sex and disease duration from diagnosis. Odds ratios > 1 indicate that higher levels of disability are associated with the presence of contrast-enhancing lesions. We report odds ratios per 5-point decrease in sSDMT and cSDMT, and per 10-m decrease in s2MWT

^a^Overall association over time, with level values on the day of the MRI

^b^Disentangled associations over time, with level values on the day of the MRI

^c^Overall association over time, with level values 6 weeks prior and 6 weeks after the MRI

^d^Disentangled associations over time, with level values 6 weeks prior and 6 weeks after the MRI

*CI* confidence interval; *MRI* magnetic resonance imaging; *sSDMT* smartphone-based symbol digit modalities test; *s2MWT* smartphone-based 2-min walk test

## Discussion

Our study shows that the digital biomarkers sSDMT and s2MWT are associated with radiological disease activity in PwRRMS, as these biomarkers significantly change in the presence of contrast-enhancing lesions. Moreover, due to weekly digital testing, we were able to demonstrate that sSDMT values from 6 weeks prior to the MRI scan showed similar relations with radiological disease activity. While standard clinical measures were also associated with radiological disease activity, we were not able to investigate their relations 6 weeks prior to the MRI, as these measures are only captured during clinical visits.

The proposed value of frequent, digital monitoring in clinical care lies in the ability to remotely capture individual functioning over extensive periods of time and in detecting subtle changes. With our hybrid model, we were able to demonstrate that the association between the sSDMT and radiological disease activity was mainly caused by variation within individuals, instead of variation between individuals, indicating that when a person’s sSDMT score decreases, this particular person has higher odds to show radiological disease activity on their MRI scan [13]. This strong association caused by variation within individuals emphasizes the potential role of the sSDMT in detecting subtle changes in functioning due to inflammation in individuals over time. Moreover, the even stronger association caused by variation within individuals when using sSDMT values from 6 weeks prior to the MRI scan might suggest that this biomarker could detect disease activity in a very early phase, possibly even before visibility on MRI. However, as we did not acquire an MRI scan at this timepoint, we were unable to eliminate the possibility that CELs were already present when the digital biomarker values changed.

Another factor to consider when interpreting these within-subject relations for the sSDMT is the possibility that a practice effect has influenced the sSDMT scores. Whereas the practice effect has not been formally investigated for high frequency sSDMT testing, research has illustrated such effects for the clinical version of the SDMT, indicating that also the digital version might be subject to these practice effects [17, 18]. This hypothesis is further supported by the increase in sSDMT level values in the first months of follow-up for a participant with radiological disease activity, as visualized in Fig. 2. Unfortunately, at the time that this study was designed, limited information was available regarding practice effects for both clinical and digital versions of the SDMT. Future studies including the sSDMT should consider these effects when designing their study protocol in order to disentangle the practice effect from the true association between this digital biomarker and radiological disease activity. Another interesting approach for future research would be to focus on the practice effect of the SDMT as a potential biomarker for cognitive functioning, instead of a confounding factor. As digital monitoring enables frequent testing, the sSDMT could be used to further explore this. That the transient dynamics of the sSDMT do not solely depend on the learning effect is supported by the development of the within-subject relation using sSDMT values from 10 weeks prior to 10 weeks after the MRI scan (Fig. 3), with the strongest relation 6 weeks prior to the MRI scan. Moreover, the diminishing relation between radiological disease activity and the sSDMT values 6 weeks after the MRI scan could suggest that when inflammatory activity subsides, sSDMT values improve. However, as we were not able to exclude the possibility that the detected CELs and thus inflammation were still present at this timepoint, this remains hypothetical as well. Future studies with the required imaging across different timepoints are necessary to further assess the transient dynamics of the sSDMT in the context of radiological disease activity.

While cognitive impairment in MS is often described as a predominantly progressive process, there are some studies implying that cognitive functions can transiently decline during active inflammation [19, 20], with some indicating the existence of isolated cognitive relapses in PwRRMS [21], supporting the relation between the sSDMT and radiological disease activity in our study. The suggestion that cognitive functions may transiently decline during the inflammatory phase might explain why our results show a stronger relation between radiological disease activity and the sSDMT, compared to the cSDMT, as the level values of the digital version are based on frequent measurements over a more extensive period of time, while the clinical version is a snapshot on the day of the study visit. These frequent sSDMT level values, which are corrected for measurement error due to physiological variation, may increase the sensitivity to change in cognition during the early inflammatory timeframe as suggested by its strong association with radiological disease activity using values from 6 weeks prior to the MRI scan. An important factor that might influence cognition and thus scoring on the sSDMT and cSDMT is fatigue. As we have not corrected for this in our analyses, this might have affected our results.

The relation between the s2MWT and radiological disease activity on the day of the MRI was caused by both variation within individuals and variation between individuals equally, with no overall association when using values from before the MRI scan. Moreover, the demonstrated relation with radiological disease activity did not diminish after six weeks. Therefore, the s2MWT appears less suitable to monitor individuals over time and less sensitive to subtle worsening in clinical performance due to inflammation, compared to the sSDMT. In line with the cognitive measures, all measures for walking ability (s2MWT, T25FW, EDSS) were associated with radiological disease activity. The absence of a relation between radiological disease activity and the s2MWT 6 weeks prior to the MRI might imply a limited role for this biomarker in detecting deterioration in clinical functioning due to disease activity in an early phase, raising the question if we should not stick to the established measures for walking ability. However, a clear advantage of the s2MWT remains that it can be administered frequently and without the necessity of a visit to the outpatient clinic. When aiming to detect subtle changes in walking ability between clinical visits, the s2MWT could be a valuable addition to the established clinical measures. Furthermore, future versions of the 2MWT with enhanced data collection and distance prediction accuracy could potentially improve its sensitivity.

A limitation of our study was the small sample size and the relatively small number of participants with CELs, resulting in less accurate estimates with broad confidence intervals and a risk of overfitting. Another limitation is the MRI slice thickness of 3 mm, instead of 5 mm according to the MAGNIMS guidelines, which may have resulted in underdetection of the number of CELs [1]. Although new T2 lesions are commonly used as a marker for radiological disease activity in clinical practice, we did not include them in our study because we were unable to define a restricted interval for their occurrence that would allow us to investigate changes in digital biomarkers. Moreover, our study showed high levels of missing data, which may introduce bias if only measurements from a selective subset of individuals remain. As no clear differences were observed between participants with and without missing digital biomarker data, this does not seem to be the case in our study. An important challenge in consistent data collection using digital biomarkers in general involves the compliance of PwRRMS [10]. This is something to take into account in the further development of these monitoring tools and in finding its place in clinical care. Ways to move forward might be to engage PwRRMS in studies on what the regular use of a digital monitoring tool in daily life means and if they are willing to adhere to weekly tests, or to further develop these tools to enable unintrusive data collection by regular smartphone use. In addition, it is of great importance for both researchers and clinicians to work together towards the clinical applicability of smartphone-based monitoring tools in clinical practice, in such a way that collected data have implications for the shared decision-making process, with the benefits of digital monitoring outweighing the burden that it might create for PwRRMS. In this context, establishing cut-off values to define meaningful changes could be of great value. Despite the limitations mentioned above and the exploratory nature of this study, we are optimistic that our results lay a solid foundation for future research on digital biomarkers in MS care and research.

### Conclusion

In conclusion, the sSDMT and s2MWT show to be capable of measuring changes in PwRRMS when inflammatory disease is detectable on MRI, with the sSDMT additionally capturing changes 6 weeks prior to the MRI. Furthermore, the diminishing association with radiological disease activity after the inflammatory phase suggests the capability of the sSDMT to capture transient dynamics. The benefit of detecting and acting on subtle clinical worsening due to disease activity in an early phase, makes a valid argument to further explore the use of these digital biomarkers in clinical practice and research.

## Supplementary Information

Below is the link to the electronic supplementary material.

Supplementary file1 (DOCX 91 KB)

## Data Availability

Data may be shared (pseudonymized) at the request of any qualified investigator for purposes of replicating procedures and results.
