# Supplementary material for: Digital monitoring of disease activity in relapsing–remitting multiple sclerosis
Source: J Neurol. 2026 Mar 6;273(3):186. doi: 10.1007/s00415-026-13685-5 (PMC12966240; doi:10.1007/s00415-026-13685-5)
Supplement: Supplementary file 1 — Supplementary file1 (DOCX 91 KB) [file 415_2026_13685_MOESM1_ESM.docx]

**Supplementary material**

*Missing data analyses*

Because of the high amount of missing data in our study, we performed a missing data analysis to determine if missing data are missing completely at random (MCAR) or not missing completely at random (not-MCAR). We did this for the sSDMT and s2MWT separately by comparing all measurements of PwMS with complete digital biomarker data on all five visits to all measurements of PwMS with missing digital biomarker data on at least one of the visits using univariate testing ^20^. Continuous variables with a skewed distribution were log transformed prior to testing. For both digital biomarkers we ran the analyses for sex, disease duration from diagnosis, EDSS, T25FW, cSDMT and NHPT. Supplementary figure 1 shows that we did not find significant differences between cases with complete sSDMT data and cases with missing sSDMT data on at least one of the five visits. Although supplementary figure 2 shows a difference in T25FW (mean difference = 0.59 seconds, p < 0.01) and NHPT scores (median difference = 1.2 seconds, p = 0.03) between complete cases and cases with missing data for the s2MWT on at least one of the five visits, the magnitude of the difference falls within the measurement error of these clinical outcomes ^21,22^. Therefore, we state that there are no relevant differences between complete cases and cases with missing data on the s2MWT. In conclusion, we classify the missing sSDMT and s2MWT data as missing completely at random (MCAR) and non-informative. In the literature it is stated that GEE-analyses may be performed without prior multiple imputation if data is MCAR and that the results might even be more robust and accurate without imputation even if the data is MAR ^23,24^. Therefore, we decided to run our analyses on the raw data.

**Supplementary figure 1.** Missing data analyses for smartphone-based Single Digit Modalities Test


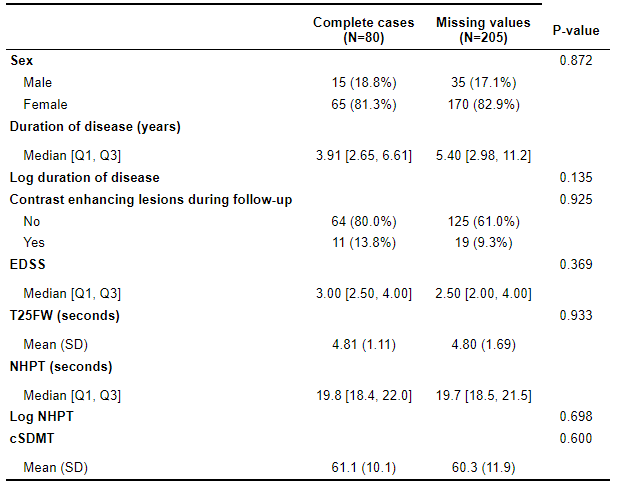


Differences in sex and the presence of contrast enhancing lesions where tested using a Chi-squared test. Differences in duration of disease, EDSS, T25FW, 9-HPT and cSDMT were tested using and independent samples t-test. Continuous variables
Abbreviations: EDSS = Expanded Disability Status Scale; T25FW = Timed 25 Foot Walk; NHPT = Nine-Hole Peg Test; cSDMT = Symbol Digit Modalities Test.

**Supplementary figure 2. Missing data analyses for smartphone-based 2 Minute Walk Test**

**
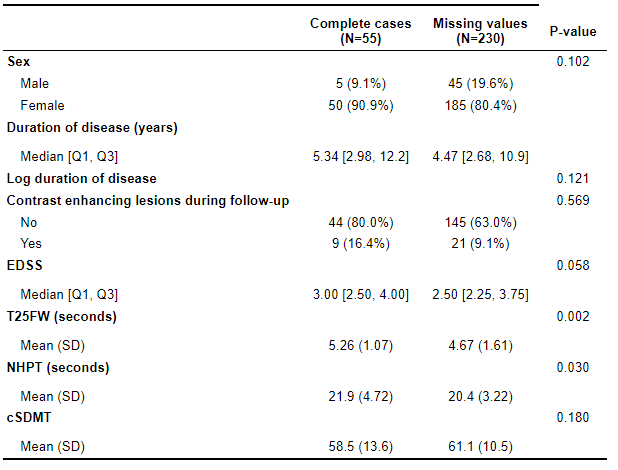
**

Differences in sex and the presence of contrast enhancing lesions where tested using a Chi-squared test. Differences in duration of disease, EDSS, T25FW, 9-HPT and cSDMT were tested using and independent samples t-test. Abbreviations: EDSS = Expanded Disability Status Scale; T25FW = Timed 25 Foot Walk; NHPT = Nine-Hole Peg Test; cSDMT = Symbol Digit Modalities Test.
